# Prospective Associations between Single Foods, Alzheimer’s Dementia and Memory Decline in the Elderly

**DOI:** 10.3390/nu10070852

**Published:** 2018-06-29

**Authors:** Karina Fischer, Debora Melo van Lent, Steffen Wolfsgruber, Leonie Weinhold, Luca Kleineidam, Horst Bickel, Martin Scherer, Marion Eisele, Hendrik van den Bussche, Birgitt Wiese, Hans-Helmut König, Siegfried Weyerer, Michael Pentzek, Susanne Röhr, Wolfgang Maier, Frank Jessen, Matthias Schmid, Steffi G. Riedel-Heller, Michael Wagner

**Affiliations:** 1 Department of Geriatrics and Aging Research, University Hospital Zurich, 8091 Zurich, Switzerland; karina.fischer@uzh.ch; 2 Centre on Aging and Mobility, University of Zurich and City Hospital Waid, 8037 Zurich, Switzerland; 3 Department of Nutrition and Food Sciences, Nutritional Epidemiology, University of Bonn, 53113 Bonn, Germany; 4 German Center for Neurodegenerative Diseases (DZNE), 53127 Bonn, Germany; steffen.Wolfsgruber@dzne.de or Steffen.Wolfsgruber@ukb.uni-bonn.de (S.W.); luca.Kleineidam@dzne.de or Luca.Kleineidam@ukbonn.de (L.K.); frank.jessen@dzne.de or frank.jessen@uk-koeln.de (F.J.); matthias.schmid@dzne.de or matthias.schmid@imbie.uni-bonn.de (M.S.); michael.Wagner@dzne.de or michael.wagner@uni-bonn.de (M.W.); 5 Department of Neurodegenerative Diseases and Geriatric Psychiatry, University of Bonn, 53105 Bonn, Germany; wolfgang.maier@ukb.uni-bonn.de; 6 Department of Medical Biometry, Informatics and Epidemiology, University Hospital Bonn, 53105 Bonn, Germany; weinhold@imbie.uni-bonn.de; 7 Department of Psychiatry, Technical University of Munich, 81675 Munich, Germany; horst.bickel@tum.de; 8 Department of Primary Medical Care, Center for Psychosocial Medicine, University Medical Center Hamburg-Eppendorf, 20246 Hamburg, Germany; m.scherer@uke.de (M.S.); meisele@uke.uni-hamburg.de (M.E.); bussche@uke.de (H.v.d.B.); 9 WG Medical Statistics and IT-Infrastructure, Institute of General Practice, Hannover Medical School, 30625 Hannover, Germany; wiese.birgitt@mh-hannover.de; 10 Department of Health Economics and Health Services Research, Hamburg Center for Health Economics, University Medical Center Hamburg-Eppendorf, 20246 Hamburg, Germany; h.koenig@uke.de; 11 Central Institute of Mental Health, Medical Faculty Mannheim/Heidelberg University, 68159 Mannheim, Germany; Siegfried.Weyerer@zi-mannheim.de; 12 Institute of General Practice, Medical Faculty, Heinrich-Heine-University Düsseldorf, 40227 Düsseldorf, Germany; Pentzek@med.uni-duesseldorf.de; 13 Institute of Social Medicine, Occupational Health and Public Health, University of Leipzig, 01403 Leipzig, Germany; Susanne.Roehr@medizin.uni-leipzig.de (S.R.); Steffi.Riedel-Heller@medizin.uni-leipzig.de (S.G.R.-H.); 14 LIFE—Leipzig Research Center for Civilization Diseases, University of Leipzig, 01403 Leipzig, Germany; 15 Department of Psychiatry, Medical Faculty, University of Cologne, 50924 Cologne, Germany

**Keywords:** food intake, gender, apolipoprotein E ε4, memory decline, cognitive decline, dementia, Alzheimer´s dementia

## Abstract

*Background*: Evidence whether single “cognitive health” foods could prevent cognitive decline is limited. We investigated whether dietary intake of red wine, white wine, coffee, green tea, olive oil, fresh fish, fruits and vegetables, red meat and sausages, assessed by a single-food-questionnaire, would be associated with either incident Alzheimer’s dementia (AD) or verbal memory decline. *Methods:* Participants aged 75+ of the German Study on Aging, Cognition and Dementia in Primary Care Patients (AgeCoDe) cohort were regularly followed over 10 years (*n* = 2622; *n* = 418 incident AD cases). Multivariable-adjusted joint modeling of repeated-measures and survival analysis was used, taking gender and Apolipoprotein E4 (*APOE* ε4) genotype into account as possible effect modifiers. *Results*: Only higher red wine intake was associated with a lower incidence of AD (*HR* = 0.92; *P* = 0.045). Interestingly, this was true only for men (*HR* = 0.82; *P* < 0.001), while in women higher red wine intake was associated with a higher incidence of AD (*HR* = 1.15; *P* = 0.044), and higher white wine intake with a more pronounced memory decline over time (*HR* = −0.13; *P* = 0.052). *Conclusion*: We found no evidence for these single foods to be protective against cognitive decline, with the exception of red wine, which reduced the risk for AD only in men. Women could be more susceptible to detrimental effects of alcohol.

## 1. Introduction

Cognitive decline and dementia are a major cause of disability and mortality in very old adults. Alzheimer’s dementia (AD) is the most common cause of dementia worldwide, which is a progressive neurodegenerative disorder that exponentially increases with age [1,2]. The disease is becoming a major public health concern and socioeconomic burden with an estimated global prevalence of about 107 million in 2050 [3]. In order to counteract this trend, modifiable lifestyle factors, such as diet, may play a pivotal role in the prevention and treatment of cognitive decline and AD [4,5]. 

In recent years, increasing interest has been devoted to the role of dietary factors as risk factors in AD and cognitive impairment [5,6]. To date, no dietary approach has been conclusively proven to protect against cognitive decline [7]. Moreover, results from different studies investigating the same dietary aspect have often been inconclusive [6]. Nevertheless, various observational and controlled intervention studies suggest that specific nutrients, foods, or overall dietary patterns may be effective [6,7,8,9,10,11]. Because nutrients are not consumed in isolation but as part of foods or a diet with possible synergistic or antagonistic interactions [12], investigating foods or overall dietary patterns rather than single nutrients seems to be a more promising approach. However, when information is only available for complex dietary patterns, it is not clear which of the foods or nutrients provided by the diet are most effective and thus should be especially included in diets of individuals at high risk of developing cognitive impairment or dementia. Moreover, investigating foods is of scientific and practical advantage because single foods, or simple combinations of foods, are the units we usually consume during the day [13]. Additionally, they are easy to understand and to implement, and they are the units most appropriate for public health purposes.

Individual foods that have been inversely linked to cognitive impairment and/or dementia include fish [6,7,8,11,14], wine [7,11,15], red wine in particular [6,7,8], olive oil [7,8,16], fruits [6,7,8,11], vegetables [6,7,8,11,17], coffee [6,18], and green tea [6,19,20,21]. By contrast, high meat intake, especially of red and processed meat products, has been shown to be positively [11,22,23], or not [24] associated with risk of dementia. However, to our knowledge, no study has investigated the association of such “cognitive health” foods with cognitive decline or dementia in a German population. In addition, compared to a number of studies on food intake and global cognition, the relationships between single foods and memory decline are understudied. These associations, however, are of interest as particularly memory is affected by AD. Also, memory tests are more sensitive than global cognition tests [25].

Furthermore, recent evidence suggests that the impact of foods on cognitive health outcomes may be modified by Apolipoprotein E ε4 genotype (*APOE* ε4) [11,26,27,28,29,30,31,32,33], a major risk factor for dementia [34,35,36] or by gender [37,38,39]. While such interactions may be biologically plausible, and could lead to personalized nutrition strategies, they are rarely studied systematically. Joint modeling (JM), i.e., the simultaneous analysis of survival (time-to-event) and repeated-measures (longitudinal) data of related processes [40], is a method which is increasingly used in medical research [41,42,43], especially when the data contains nonrandom dropouts, which is common in a study including the elderly. 

To the best of our knowledge, JM has not been used to investigate the association between food intake, cognitive decline, and incidence of dementia in the elderly.

The aim of the present study was to utilize JM to investigate whether dietary intake of commonly eaten and supposed “cognitive health” foods were longitudinally associated with incidence of AD or memory decline in participants of the German Study on Aging, Cognition and Dementia in Primary Care Patients (AgeCoDe) cohort. In addition, we analyzed whether these associations were modified by gender or *APOE* ε4 status.

## 2. Methods

### 2.1. Study Design and Participants

The current data are from the German Study on Ageing, Cognition and Dementia in Primary Care Patients (AgeCoDe and Needs, health service use, costs and health-related quality of life in a large sample of oldest-old primary care patients (AgeQualiDe). The study is a German multicenter and general practitioner (GP) registry-based prospective cohort study on early detection and prediction of mild cognitive impairment and dementia in elderly primary care patients starting in 2003. Enrolled participants were primary care patients aged 75 years or older living in the urban areas of the six German cities Bonn, Düsseldorf, Hamburg, Leipzig, Mannheim, or Munich. The recruitment and baseline visits were conducted between January 2003 and November 2004. Since then, eight follow-up (FU) visits (with an 18-month interval between each FU) were completed up to the time of the present study. Selection and sampling of the participants have been described previously [44,45]. Briefly, participants were recruited by 138 GPs connected to the respective study sites. Of the GP population of 22,701 persons, a total of 10,850 were eligible for inclusion in the study. Of those, 6619 persons were randomly selected to be invited to participate in the study. Inclusion criteria were age above 75 years, absence of dementia [46], and at least one personal contact with the GP during the past year. Exclusion criteria were consultations only via home visits, residence in a nursing home, prevailing severe illness with an expected fatal outcome within the next three months, insufficient German language skills, blindness or deafness, inability to provide an informed consent, and not being a regular patient of the participating GP. Of the 6619 invited persons, 3327 persons consented to enrollment and were investigated at baseline. All baseline assessments were performed by trained investigators (physicians, psychologists, gerontologists) at the participants’ homes and included structured clinical interviews comprising sociodemographic and anthropometric information, neuropsychological tests, current physical and mental health, and psychosocial and lifestyle factors. The same personal structured interviews and neuropsychological assessments were conducted in subsequent FUs at 18-month intervals. For participants who could not be interviewed personally at any FU visit, informant-based information was obtained. In such a case, participants were excluded from further FUs. The study was approved by the local ethics committees of the six participating centers, and all participants gave their written informed consent to the study.

### 2.2. Analytical Samples

**Figure 1:**
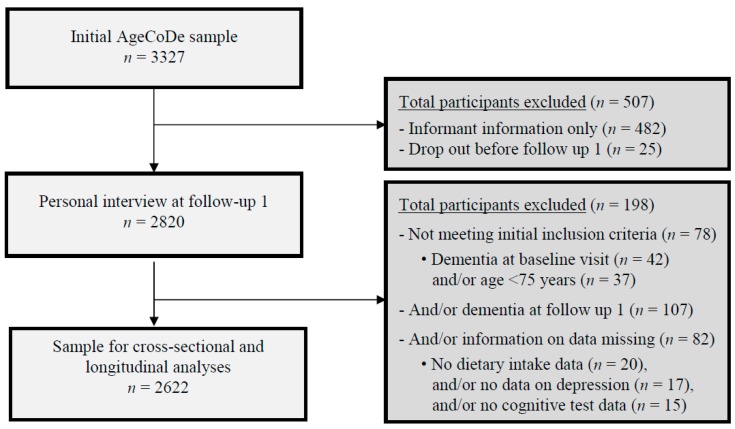
Flow chart of the participants included in the AgeCoDe study. Abbreviations: AgeCoDe, German Study on Aging, Cognition and Dementia in Primary Care Patients.

For the present study, we used data of participants who attended the first follow-up (FU-1) visit because information on food intake was collected at FU-1. Of the 3327 participants investigated at baseline, we excluded participants with FU-1 informant-based information only (*n* = 482) and participants that were lost to follow up before the first follow up visit (*n* = 25), resulting in a sample of 2820 participants at FU-1. From these 2820 participants, we further excluded in total 198 participants who, at baseline, received a study diagnosis of dementia (*n* = 42) or were aged below 75 years (*n* = 37; falsely classified as 75 years or older in the study selection process) as well as those participants with dementia at FU-1 (*n* = 107), and/or those participants whose dietary intake data (*n* = 20) and/or data on depression (*n* = 17), and/or cognitive data (data on the Consortium to Establish a Registry for Alzheimer’s Disease (CERAD); *n* = 15) was not available at FU-1. Consequently, the sample of non-demented participants with complete cognitive test data at FU-1 included 2622 participants. The exclusion of participants of the present study is shown in a flow chart (Figure 1).

### 2.3. Dietary Assessment

At FU-1, dietary intake of foods was assessed using a short and concise 8-item “cognitive health” food intake screener developed by the AgeCoDe study group. Participants were asked how often they usually consumed fresh fish (not canned), olive oil, fruits and vegetables (excluding potatoes), meat and sausages, red wine, white wine, green tea, and coffee. For each food item, options to answer were (1) “never”; (2) “less than once a week”; (3) “once a week”; (4) “several times per week”; (5) and “each day”, resulting in an intake score (range 0–4) for each food item.

### 2.4. Assessment and Diagnosis of Alzheimer’s Dementia

Participants were assessed with the SIDAM, an established structured interview for the diagnosis of dementia of the Alzheimer type, multi-infarct dementia and dementias of other aetiology [47,48]. AD was diagnosed by consensus of the interviewing investigator and an experienced geriatrician or geriatric psychiatrist according to DSM-IV and ICD-10 criteria that are implemented as a diagnostic algorithm in the SIDAM [47,48]. This algorithm comprises cognitive impairment, as defined by the total SIDAM cognitive score (SISCO, scoring 0–55 with a higher score indicating a better performance as the sum of the MMSE score (0–30) and 25 additional items) and impairment of activities of daily living (ADL) as defined by a score of at least two points on the SIDAM-ADL-scale. For dementia, the etiological diagnosis of AD was established according to the National Institute of Neurological and Communicative Disorders and Stroke and the Alzheimer’s Disease and Related Disorders Association (NINCDS-ADRDA) criteria for probable AD [49]. For the diagnosis of vascular dementia, that is, in case of evidence for cerebrovascular events (Hachinski–Rosen Scale, medical history) and a temporal relationship between the cerebrovascular event and the occurrence of cognitive decline, the National Institute of Neurological Disorders and Stroke and Association Internationale pour la Recherché et l´Enseignement en Neurosciences (NINDS-AIREN) criteria were used [50]. Mixed dementia was diagnosed in cases of cerebrovascular events without temporal relationship to cognitive decline. Dementia diagnosis in participants who were not personally interviewed was based on the Global Deterioration Scale [51] and the Blessed Dementia Rating scale [52]. A score of 4 or higher on the Global Deterioration Scale was used as the criterion for the dementia diagnosis. In these cases, an etiological diagnosis was established if the information provided was sufficient to judge etiology according to the above-named criteria. For statistical analyses AD and mixed dementia were combined into one AD group.

### 2.5. Neuropsychological Assessment

In addition to the cognitive scale in the SIDAM, participants were assessed with subtests of the CERAD neuropsychological assessment battery, which was designed to cover the cognitive domains most commonly affected in AD dementia [53]. The administered CERAD subtests varied across follow-ups, but always included the immediate and delayed verbal memory subtests of the CERAD. In the present study, we used the ten-item Word List Immediate Recall subtest (score 0–30), the ten-item Word List Delayed Recall subtest (score 0–10), and the ten-item Word List Recognition subtest (scored by subtracting False Alarms from Hits; score 0–10) to quantify objective episodic memory performance. Higher scores indicate a better memory performance in all subtests. From these subtests we created one total memory score by standardizing a single subtest to percentages of its possible maximum score (i.e., dividing the respective score by its maximum score and multiplying the result by 100) and averaging the resulting three standardized subtest scores to a total CERAD memory score (range 0–100).

### 2.6. Assessment of Covariates

Information on sociodemographic, clinical, psychometric, and lifestyle data was collected at the baseline assessment or FU-1 during structured interviews at the participants’ homes. For the present study, education was classified into three levels (low, middle, and high) based on the Comparative Analysis of Social Mobility in Industrial Nations classification system [54]. For genetic and blood biomarker analyses blood samples were drawn from each participant at the attending GP practice. Leucocyte DNA was isolated using the Qiagen blood isolation kit according to the manufacturer´s instructions (Qiagen, Hilden, Germany). ApoE genotyping was performed according to standard procedures [55]. Participants were grouped into those with at least one *APOE* ε4 allele (homo- and heterozygous carriers; positive *APOE* ε4 status) and those without an ε4 allele (non-carriers, negative *APOE* ε4 status). Height and weight measured at FU-3 were used as a proxy for height and weight at FU-1. BMI was calculated based on weight divided by height squared. Smoking status was assessed at the baseline visit and used as a proxy for FU-1. Smoking was divided into three categories as never smoker, former smoker, and current smoker. Assessment of physical activity was evaluated at FU-1 based on Verghese et al. [56] with small modifications. In brief, participants reported the frequency of usual engagement in each of the six physical activities: bicycling, walking, swimming, gymnastics, chores/gardening, and a category of other physical leisure activities (e.g., bowling, jogging, or golfing) using five possible options to answer: (1) “each day”; (2) “several times per week”; (3) “once a week”; (4) “less than once a week”; and (5) “never”. For the present study, the five frequency categories whether the participant usually engaged in one of the six physical activities were collapsed into three categories with the following scoring: “each day” and “several times per week” = 2; “once a week” = 1; and “less than once a week” and “never” = 0. For each participant, these values (0, 1, or 2) were summed up across the six activities to a total physical activity score (range 0–12). Depressive symptoms were assessed by using the short 15-item version of the Geriatric Depression Scale [57], with a cut-off point of 6 or more used to indicate depressive symptomatology [58]. Regarding comorbidities, we used a modified score of the Charlson comorbidity index (CCI) [59] as a proxy for disease status. The diseases included in our modified CCI score based on GP record information and comprised: myocardial infarction, congestive heart failure, peripheral vascular disease, cerebrovascular disease, rheumatism, diabetes, liver disease, and chronic kidney disease. All diseases were coded whether the disease was present or not (yes = 1; no = 0), with chronic kidney disease counting double according to the original CCI score. For each participant, these values (0 or 1) were summed up across the eight diseases resulting in our total modified CCI score (range 0–9).

### 2.7. Statistical Analysis

Participant characteristics at FU-1 were cross-sectionally analyzed for the total population and stratified by gender and *APOE* ε4 status. Differences in participant characteristics between men and women or between *APOE* ε4 carriers and non-carriers were examined using Student´s *t*-tests for continuous variables and Chi-square tests for categorical variables.

Joint modeling [43] of survival data (with time-to-event dichotomous variable “incidence of AD” as the response variable) and longitudinal data (with the continuous variable “memory decline” as the response variable) was used to investigate the longitudinal associations between intake of foods and incidence of AD together with memory decline. JM combines a Cox proportional hazards sub-model and a linear-mixed-effects repeated-measures sub-model by linking the respective random effect terms, thereby accounting for incomplete observed time-varying covariate information and possible informative dropout. For the linear-mixed-effects model part of the analyses, participant-specific random intercept terms and random time slopes were incorporated into the models. To examine whether the intake of the single foods modified memory over time, i.e., velocity, we included interaction terms between each single food and time in the fixed-effects structure. Results of JM are expressed in terms of hazard ratios (for time-to-event submodels, with 95% confidence intervals) and coefficient estimates (for linear mixed-effects submodels, with 95% confidence intervals). 

Confounders were selected based on published literature. Five confounders (weight, *APOE* ε4 status, physical activity, hypercholesterolemia, and the modified CCI) contained missing values (Supplementary Table S1). The percentages of missing values ranged from 0.2% (weight) to 3.6% (*APOE* ε4 status and modified CCI). To account for potential attrition bias, multiple imputation was used by creating ten different possible copies of the original dataset, in which the missing values were substituted by imputed values (Table 1). These imputed values were calculated from their predictive distribution based on the observed data (35). Combined results of the created datasets (*n* = 10) were then pooled in a separate pooled dataset, to account for the uncertainty about the missing values. A description of the procedure is reported (Supplementary Table S2). All models were run using imputed data.

Models were adjusted for socio-demographic factors: age, gender, BMI, education, and *APOE* ε4 status (model 1); or additionally to model 1, for the lifestyle factors smoking status and physical activity, depression, hypercholesterolemia, and a modified CCI score (model 2). We did not adjust for other single foods because these foods would not account for all dietary factors that affect memory decline. Moreover, to account for participants´ cognitive status at FU-1, the linear mixed model included the CERAD memory FU-1 score as the first repeated measure.

We aimed to investigate effect modification by *APOE* ε4 status and gender. We tested for significant effect modification of the longitudinal associations between individual foods and all outcome variables by *APOE* ε4 status and gender, by including a multiplicative interaction term (food intake frequency × gender × time, or food intake frequency × *APOE ε4* status × time) in model 2. In the case of a significant interaction term (*P* < 0.10) for the AD sub-model and/or the memory decline sub-model of the JM, we stratified the analysis by *APOE* ε4 status or gender to reveal the strength and direction of the associations in the individual subgroups.

*P* values are two-sided, and *P* < 0.05 was considered statistically significant. IBM SPSS Statistics for Windows (Release 21, IBM Corporation, Armonk, NY, USA) was used for descriptive analyses at FU-1; and R.3.0.1 under R studio was used for the longitudinal JM analyses.

## 3. Results

### 3.1. Participant Characteristics

Participant characteristics are presented for the total study population (81.2 ± 3.4 years, 65.3% women, 4.5 ± 2.8 years to develop AD) as well as by gender and *APOE ε4* status (Table 1). As compared to men, women were older (81.3 vs. 80.9 years, *P* = 0.001), had lower BMI (25.7 vs. 26.1 kg/m^2^, *P* = 0.003), lower physical activity (*P* = 0.048), lower educational level (*P* < 0.001), lower tendency to smoke (*P* < 0.001), higher frequency of Mild Cognitive Impairment (MCI) (18.4% vs. 13.3%, *P* = 0.001), more often prevalent depression (12.7% vs. 8.8%, *P* = 0.002), and less often comorbidities (*P* < 0.001) than men, but higher CERAD memory score (73.0 vs. 69.3, *P* < 0.001) and more years to censoring (6.0 vs. 5.7, *P* = 0.047) than men. As compared to *APOE* ε4 non-carriers (Table 1), *APOE* ε4 carriers had a lower BMI (25.6 vs. 25.9 kg/m^2^, *P* = 0.048), a higher frequency of MCI (21.7% vs. 15.4%, *P* = 0.001) and hypercholesterolemia (57.5% vs. 52.7%, *P* = 0.014), together with lower CERAD memory scores (69.4 vs. 72.3, *P* < 0.001) and fewer years to censoring (5.6 vs. 6.0 years, *P* = 0.021).

Over the 10-year FU, in the total study population (*n* = 2622), 418 participants developed AD. Stratified by gender, 107 men and 311 women developed AD, whereas stratified by *APOE ε4* status, 126 *APOE* ε4 carriers and 292 *APOE* ε4 non-carriers developed AD over the period of 10 years.

Food intake frequencies of the study participants are presented for the total population as well as by gender and *APOE* ε4 status (Table 2). Food intake frequencies differed between men and women, with women reporting a more frequent consumption of fruits and vegetables, and men reporting a more frequent consumption of fresh fish, olive oil, meat and sausages, red wine, white wine and green tea (Table 2). Food intake frequencies between *APOE* ε4 carriers and *APOE* ε4 non-carriers did not differ significantly for any food item (Table 2).

### 3.2. Longitudinal Associations Between Food Intake and Incident AD or Memory Decline in JM

In the JM of AD and memory decline (Table 3 and Table S3), higher red wine intake was significantly associated with lower incidence of AD in both model 1 and model 2 (model 2: *HR* = 0.92; 95% CI: (0.85, 0.99); *P* = 0.045). No significant associations between higher intakes of the single foods and AD and memory decline were observed in these JM. Furthermore, additional adjustment for food combinations, excluding the food under investigation, did not change the results.

We found evidence for effect modification of the association between intake of red wine and incident AD by gender, as well as of the association between intakes of fruits and vegetables, red wine, meat and sausages, white wine, and coffee and incident AD by *APOE* ε4 status (Table 3). In addition, we found evidence for effect modification of the association between intakes of olive oil and white wine and memory decline by gender (Table 3).

We performed stratified analyses of JM of AD and memory decline only when there was evidence for effect modification (*P*_interaction_ < 0.10) by gender or *APOE* ε4 status (Table 3 and Table 4).

Stratified by gender (Table 4**,** data for model 1 not shown), higher red wine intake was associated with a lower incidence of AD among men (model 2: *HR* = 0.82; 95% CI: (0.74, 0.92); *P* < 0.001), whereas higher red wine intake was associated with a higher incidence of AD among women (model 2: *HR* = 1.15; 95% CI: (1.00, 1.32); *P* = 0.044). In addition, we observed that higher white wine intake and higher olive oil intake were borderline significant associated with a more rapid decline of memory decline in women (model 2: −0.13; 95% CI: (−0.26, 0.001); *P* = 0.052, and −0.08; 95% CI: (−0.16, 0.01); *P* = 0.065, respectively). Stratified by *APOE ε4* status (Table 4**,** data for model 1 not shown), higher white wine intake was associated with a higher incidence of AD among *APOE* ε4 carriers (*HR* = 1.21; 95% CI: (1.01, 1.46); *P* = 0.044 for model 2). No significant associations were observed between higher fruits and vegetables or meat and sausages intakes and incident AD, and higher coffee intake and memory decline.

## 4. Discussion

To our knowledge, our study is the first to investigate the association between dietary food intake and incident AD and memory decline in a German population. Overall, we found that higher red wine intake was associated with lower incidence of AD over a 10-year FU. Interestingly, these and various other associations between intakes of foods (especially red wine and white wine) and incident AD or memory decline were modified by gender or *APOE ε4* status. 

### 4.1. Red Wine and White Wine 

Red wine, more than white wine, was consumed at least once per week by about 40% of men and 20% of women in our sample. We found that higher red wine intake was associated with a lower incidence of AD. However, stratified analyses revealed that this was only true among men, as in women we rather found an increased risk for incident AD. Consistent with this, we also found an association between higher white wine intake and a more rapid memory decline in women. Epidemiological studies suggest that moderate consumption of red wine may prevent or slow age-related neurodegenerative diseases [15,60,61]. Red wine has been frequently studied with regard to cognitive decline because red grapes are one of the richest sources of polyphenols, such as resveratrol, quercetin, and catechins, that may counteract cognitive decline and AD in a multi-target manner [60,62,63]. However, it is not clear if the effect of red wine intake can be attributed to these nutrients. For example, Weyerer et al. (2011) observed an association between light to moderate alcohol intake (regardless of type of alcoholic beverages) and decreased incidence of AD [64].

Our findings could, thus, be ascribed to the alcohol content of red and white wine. Whereas in several epidemiological studies light-to-moderate drinking of alcoholic beverages has been shown to be protective against cognitive decline and AD [65], higher daily intakes or abuse of alcohol have also been shown to be detrimental for brain function due to an U-shaped dose-response relationship [66,67]. Furthermore, higher white wine intake was associated with an increased risk of AD among *APOE* ε4 carriers. These findings are in line with two literature reviews concluding that the effects of moderate wine consumption are more likely among *APOE* ε4 non-carriers [65,68].

### 4.2. Coffee 

In our German study population, higher coffee intake was not associated with memory decline or incident AD despite an indication for effect modification by *APOE* ε4 status regarding memory decline. Coffee consumption may affect cognitive functions due to antioxidant, anti-inflammatory, or neuroprotective properties of phytochemicals found in coffee, including significant amounts of chlorogenic acid and caffeine [69,70,71,72]. In line with our findings, a recent meta-analysis of eleven prospective studies did not find an association between coffee consumption and measures of cognitive decline. However, a reduced risk for AD of high versus low intakes of coffee was reported [18]. To our knowledge, two other studies investigated effect modification by *APOE* ε4 status for the risk of AD or dementia related to coffee, both studies found no significant P values for interaction [73,74]. Eskelinen et al. (2009) reported the association between moderate coffee consumption and a lower risk for dementia in both *APOE* ε4 carriers and non-carriers [73].

### 4.3. Olive Oil 

Olive oil is not so common in the German diet as in Mediterranean countries, with only about 50% of our sample consuming it at least once per week. We observed no association between high olive oil intakes and incident AD or memory decline. If any, there was a trend towards higher olive oil consumption to be related with stronger memory decline in women, contrary to expectations. Berr et al. (2009) showed that intensive olive oil intake was associated with lower odds of cognitive deficit in visual memory and verbal fluency and decline in visual memory [16]. However, most participants had a moderate to intensive consumption. In addition, the PREDIMED-NAVARRA Randomized Control Trial observed, in participants who were supplemented extra-virgin olive oil in combination with the Mediterranean diet a better cognitive function in comparison with a control diet, however, no associations were found for most cognitive domains [75]. Compared to the omega-3 fatty acids docosahexaenoic acid (DHA) and eicosapentaenoic acid (EPA) present in fish oil that play a pivotal role in brain functions [76], oleic acid, linoleic acid, and palmitic acid present in olive oil do not have specific roles in brain function. 

### 4.4. Fruits and Vegetables

Fruits and vegetables have been widely studied in relation to global cognitive and memory decline [17,77,78]. We observed no associations between higher fruits and vegetables intake and our assessed outcomes. This is in line with a systematic review of cohort studies [17], reporting that those studies that analyzed fruits and vegetables combined, did not find an association with global cognition, while studies analyzing fruits and vegetables separately found an inverse association with vegetables only, indicating that evidence for a protective role of fruit consumption in global cognition is insufficient [17].

### 4.5. Meat and Sausages 

Meat and sausage products have not been widely studied with regard to cognitive decline. In our study, we did not observe an association of higher meat and sausages intake with incident AD or memory decline, although we observed an interaction with *APOE* ε4, possibly indicating that *APOE* ε4 carriers with a higher intake of meat and sausages are at increased risk for AD. Single studies have reported that high meat intake, especially red and processed meat products, investigated individually or as part of an “unhealthy” dietary pattern were positively [11,22,23,79] or not [24] associated with cognitive decline. Concerns with regard to meat products are mainly related to the saturated fatty acids in animal fats as well as the heme iron in red meat, which are both risk factors of vascular disease [11,80]. Otherwise, meat provides high-quality protein [81] and other essential nutrients and functional components [82] with possible benefits for cognitive functioning.

### 4.6. Fresh Fish 

We did not find a significant association between fresh fish intake and of incident AD or memory decline. Seafood and fish intake, rich in brain-protective nutrients, including *n*-3 fatty acids [83], have been repeatedly associated with better cognitive function and reduced cognitive decline or lower incidence of dementia, including AD [14,84,85]. Possible reasons that we did not observe a significant association between fresh fish intake and incident AD and memory decline in our study could be that in Germany about one third of consumed fish is freshwater fish, providing no meaningful amounts of omega-3 fatty acids as compared to sea fish. Moreover, when assessing “fresh fish” only, we might not have captured all intakes of fish. 

### 4.7. Green Tea 

In contrast to our study, other cohort studies in elderly Asian adults reported less cognitive decline with higher green tea intake [21,86] rich in antioxidant, anti-inflammatory, and neuroprotective phytochemicals such as flavonoids, catechins [87,88,89], and caffeine [71,72]. In our study higher green tea intake were not significantly associated with incident AD or memory decline. This opposite finding in our German cohort where green tea is not a traditional, but a modern “health food”, may be explained by the ethnic and genetic background resulting in different metabolic responses to green tea among Asian and Caucasian individuals [90].

### 4.8. Strengths and Limitations

Our study has several important strengths. First, the large multi-center GP-based sample that allowed investigating individuals across Germany and the long observation period of 10 years. Second, standardized assessments of cognitive function were performed every 18 months, allowing for the analysis of decline trajectories and for the early detection of incident cases with dementia. Third, most effects hold true after adjusting for a range of important confounders. Fourth, we made use of the JM statistical technique to make optimal use of the available information on repeated CERAD memory measures and incident AD, with memory decline being linked to AD. An important strength of analyzing these outcomes in JM simultaneously is to increase precision, as observed in previous studies [41,42,43]. Furthermore, inclusion of dropout time into the model may help to account for unbiased estimates of cognitive decline in the presence of missing data and inclusion of the information of longitudinal cognitive assessments may also help to address potentially informative censoring in the survival part of the model [43].

On the other hand, our study also has limitations. Most important, we were limited to the eight predefined food groups included in the cognitive health screener as we were not able to assess dietary food intake using a more extensive food frequency questionnaire. In addition, some promising food groups in relation to cognitive health, such as green leafy vegetables, berries, and fatty fish, could not be assessed as they were collapsed into one ‘fruits and vegetables’ group or one ‘fresh fish’ group. Finally, we cannot fully exclude some reverse causation bias due to changes in dietary habits some time before the onset of dementia. However, the fact that results remained virtually identical for red wine intake after excluding subjects with MCI (HR = 0.92 vs HR = 0.93) makes such a reverse causation bias less likely. Given the long build-up of AD pathology, longitudinal dietary assessments starting in mid-life would be needed to address this issue.

Moreover, dietary assessment by our food intake screener may have been subject to measurement error and bias: systematic error because we were not able to adjust for total energy intake; recall bias because participants may not have accurately remembered their food intakes; non-differential misclassification possibly leading to bias towards the null [91]; and selection or survival bias because we investigated elderly participants, increasing the likelihood that cognitively normal individuals with comorbidities in an advanced stage were too sick to be enrolled in the AgeCoDe study at baseline or died before the age of 75 years. However, similar short food intake screeners have been shown to be valid for assessing intake of specific food items in older adults [92,93]. In addition, these screeners reduce the burden for respondents and interviewers and provide a reasonably accurate ranking of intake, similar to that of a full-length dietary questionnaire [94]. While the methodological limitations of our food intake screener may have introduced noise to the resulting data, this does not compromise the significance of the main and interaction effects we found. Furthermore, based on the results observed in previous studies [39,75,95,96], we expected small effect sizes in the associations between dietary food intake and outcomes of cognitive decline. A limitation of the analysis strategy used here is that it did not enforce strict type 1 error control by application of correction procedures for multiple testing. However, all single foods studied here had some prior evidence for being associated with dementia, and were not studied in a shotgun manner. 

## 5. Conclusions

In conclusion, we found no evidence for the single foods studied to be protective against memory decline and AD, with the exception of red wine, which reduced the risk for AD only in men, while increasing it in women. Women appear to be more susceptible to detrimental effects of alcohol in general, as they experienced also a steeper memory decline with higher white wine intake. We also found some evidence for effect modification by *APOE* ε4, with hazard ratios for incident AD being consistently higher in *APOE* ε4 carriers for several food items studied. This should motivate further studies regarding effect modification in nutritional epidemiology, as a step towards personalized dietary recommendations in old age.

## Figures and Tables

**Table 1:** Participant characteristics of the AgeCoDe cohort at follow-up 1.

Participant Characteristics	Total Population (*n* = 2622)	Men (*n* = 910)	Women (*n* = 1712)	*P*	*APOE* ε4 Carriers (*n* = 551)	*APOE* ε4 Non-Carriers (*n* = 2071)	*P*
Age (years)	81.2 ± 3.4	80.9 ± 3.4	81.3 ± 3.4	0.001	80.9 ± 3.3	81.2 ± 3.5	0.035
Female (*n* (%))	1712 (65.3)	-	-	-	358 (65.1)	1354 (65.3)	0.886
BMI (kg/m^2^)	25.9 ± 3.3	26.1 ± 2.8	25.7 ± 3.5	0.003	25.6 ± 3.1	25.9 ± 3.4.	0.048
*APOE* ε4 status (*n* (%))	551 (21.0)	193 (21.2)	358 (20.9)	0.886	-	-	-
Education (*n* (%))				<0.001			0.164
Low	1594 (60.8)	483 (53.1)	1111 (64.9)		327 (59.3)	1267(61.2)	
Middle	723 (27.6)	220 (24.2)	503 (29.4)		168 (30.5)	555 (26.8)	
High	305 (11.6)	207 (22.7)	98 (5.7)		56 (10.2)	249 (12.0)	
Physical activity (*n* (%))				0.048			0.252
Low (0 ≥ 3)	833 (31.8)	277 (30.4)	556 (32.5)		161 (29.2)	672 (32.5)	
Middle (3 ≤ 5)	897 (34.2)	296 (32.5)	601 (35.1)		204 (37.0)	693 (33.5)	
High (5–11)	892 (34.0)	337 (37.0)	555 (32.4)		186 (33.8)	706 (34.0)	
Smoking (*n* (%))				<0.001			0.561
Never	1307 (49.8)	178 (19.6)	1129 (66.0)		277 (50.3)	1030 (49.7)	
Past	1125 (42.9)	662 (72.7)	463 (27.0)		229 (41.5)	896 (43.3)	
Current	190 (7.3)	70 (7.7)	120 (7.0)		45 (8.2)	145 (7.0)	
MCI (*n* (%))	436 (16.6)	121 (13.3)	315 (18.4)	0.001	119 (21.7)	318 (15.4)	0.001
Hypercholesterolemia (*n* (%))	1408 (53.7)	446 (49.0)	962 (56.2)	0.094	317 (57.5)	1091 (52.7)	0.014
Depression (*n* (%))	298 (11.4)	80 (8.8)	218 (12.7)	0.002	67 (12.1)	231 (11.2)	0.345
Modified CCI score (0–6) (*n* (%))				<0.001			0.215
Score 0–2	1866 (71.2)	585 (64.3)	1281 (74.8)		408 (74.1)	1458 (70.4)	
Score 3–4	666 (25.4)	284 (31.2)	382 (22.3)		124 (22.5)	542 (26.2)	
Score 5–6	90 (3.4)	41 (4.5)	49 (2.9)		19 (3.4)	71 (3.4)	
CERAD memory (score 0–100)	71.7 ± 13.0	69.3 ± 12.8	73.0 ± 13.0	<0.001	69.4 ± 13.6	72.3 ± 12.8	<0.001
Time to develop AD (years)	4.5 ± 2.8	4.2 ± 2.7	4.6 ± 2.8	0.133	4.2 ± 2.7	4.6 ± 2.8	0.183
Time to censoring (years)	5.9 ± 3.3	5.7 ± 3.3	6.0 ± 3.3	0.047	5.6 ± 3.3	6.0 ± 3.3	0.021

Based on imputed data. Data (*n* = 2622) are means (±standard deviation or *n* (%). *P* < 0.05 was considered statistically significant. Abbreviations: AgeCoDe, German Study on Aging, Cognition and Dementia in Primary Care Patients; AD, Alzheimer´s dementia; *APOE* ε4, apolipoprotein E ε4 allele; BMI, body mass index; CCI, Charlson comorbidity index; MCI, mild cognitive impairment; CERAD; Consortium to Establish a Registry for Alzheimer’s Disease.

**Table 2:** Food intake frequencies (%) in participants of the AgeCoDe cohort at follow-up 1.

Food Intake Frequencies (%)	Total Population	Men	Women	*APOE* ε4	
				Carriers	Non-Carriers
	(*n* = 2622)	(*n* = 910)	(*n* = 1712)	(*n* = 551)	(*n* = 2071)
Fruits and vegetables ^a^					
Never	0.3	**0.4**	**0.2**	0.2	0.3
<1 time/week	0.2	**0.3**	**0.1**	-	0.2
1 time/week	0.5	**1.0**	**0.2**	0.4	0.5
Several times/week	14.8	**18.5**	**12.9**	17.1	14.2
Every day	84.2	**79.8**	**86.6**	82.4	84.7
Fresh fish					
Never	7.2	**6.7**	**7.5**	6.7	7.3
<1 time/week	30.0	**26.6**	**31.8**	31.2	29.6
1 time/week	44.9	**49.0**	**42.7**	42.3	45.6
Several times/week	17.8	**17.4**	**18.0**	19.6	17.3
Every day	0.2	**0.3**	**0.1**	0.2	0.1
Olive oil					
Never	36.3	**32.1**	**38.6**	38.3	35.2
<1 time/week	12.2	**13.0**	**11.9**	10.3	12.5
1 time/week	8.2	**7.7**	**8.5**	9.1	7.8
Several times/week	32.2	**35.8**	**30.2**	32.7	31.5
Every day	11.1	**11.4**	**10.9**	9.6	13.0
Meat and sausages ^a^					
Never	1.1	**0.7**	**1.3**	1.5	1.0
<1 time/week	2.6	**1.0**	**3.4**	2.7	2.5
1 time/week	8.5	**3.5**	**11.2**	9.6	8.2
Several times/week	51.3	**45.9**	**54.1**	49.9	51.6
Every day	36.6	**48.9**	**30.1**	36.3	36.7
Red wine					
Never	52.2	**38.6**	**59.4**	52.3	52.1
<1 time/week	20.4	**22.0**	**19.6**	21.4	20.2
1 time/week	9.2	**11.3**	**8.1**	9.1	9.3
Several times/week	10.7	**16.7**	**7.5**	9.8	10.9
Every day	7.5	**11.4**	**5.4**	7.4	7.5
White wine					
Not at all	64.4	**51.4**	**71.3**	61.9	65.1
<1 time/week	20.6	**25.3**	**18.2**	22.0	20.3
1 time/week	6.1	**9.6**	**4.3**	7.3	5.8
Several times/week	7.0	**11.1**	**4.8**	6.9	7.0
Every day	1.9	**2.6**	**1.5**	2.0	1.8
Coffee					
Never	13.2	13.0	13.3	13.1	13.2
<1 time/week	5.2	5.9	4.8	4.2	5.5
1 time/week	3.4	3.8	3.1	3.1	3.5
Several times/week	6.6	7.3	6.3	6.2	6.7
Every day	71.6	70.0	72.5	73.5	71.1
Green tea					
Never	67.7	71.1	65.9	69.3	67.3
<1 time/week	12.6	**12.0**	**12.9**	11.1	12.9
1 time/week	5.0	**3.2**	**6.0**	4.4	5.2
Several times/week	8.2	**5.8**	**9.5**	7.8	8.4
Every day	6.4	**7.9**	**5.6**	7.4	6.2

Data (*n* = 2622) are (%). Abbreviations: AgeCoDe, German Study on Aging, Cognition and Dementia in Primary Care Patients; *APOE* ε4, apolipoprotein E ε4 allele. ^a^ After collapsing categories to have at least *n* = 5 per category, chi-square tests revealed significant differences in the frequency (percentages are bolded) of dietary intakes for fruits and vegetables, fresh fish, olive oil, meat and sausages, red wine, white wine and green tea between men and women only. *P* < 0.05 was considered statistically significant.

**Table 3:** Longitudinal joint modeling associations between food intake and incident AD and memory decline over a 10-year follow-up period.

Associations between Food Intake and Incident AD or Memory Decline	*HR* (95% CI) for Incident AD and UnstandardizedRegression Coefficients (95% CI) for Memory Decline		Significant *P*-Values for Interaction (*P* < 0.10)	
	Model 2		Gender	*APOE* ε4 status
Incident AD				
(survival sub-model)	*HR* (95%CI)	*P*		
Fruits and vegetables	1.08 (0.80; 1.46)	0.609	-	0.085
Fresh fish	0.98 (0.87; 1.11)	0.754	-	-
Olive oil	1.00 (0.93; 1.07)	0.969	-	-
Meat and sausages	1.09 (0.94; 1.26)	0.236	-	0.083
Red wine	0.92 (0.85; 0.99)	0.045	0.001	-
White wine	1.00 (0.91; 1.12)	0.875	-	0.074
Coffee	0.97 (0.90; 1.04)	0.338	-	-
Green tea	0.94 (0.86; 1.02)	0.129	-	-
Memory decline				
(repeated-measures sub-model)	*B* (95%CI)	*P*		
Fruits and vegetables	0.10 (−0.14; 0.33)	0.408	-	-
Fresh fish	−0.03 (−0.14; 0.08)	0.610	-	-
Olive oil	−0.03 (−0.09; 0.04)	0.388	0.064	-
Meat and sausages	0.01 (−0.11; 0.14)	0.845	-	-
Red wine	−0.04 (−0.11; 0.03)	0.302	-	-
White wine	−0.03 (−0.12; 0.06)	0.494	0.085	-
Coffee	−0.02 (−0.08; 0.05)	0.241	-	0.056
Green tea	0.02 (−0.06; 0.09)	0.681	-	-

Based on imputed data (*n* = 2622). Model 2 was adjusted for age, gender, BMI, education, *APOE* ε4 carrier status, smoking status, physical activity score, depression, hypercholesterolemia, and a modified CCI score (for model 1 see Table S3 in the Supplementary Materials part). *P* < 0.05 was considered statistically significant. Abbreviations: HR, hazard ratio; AD, Alzheimer’s dementia; BMI, body mass index.

**Table 4:** Analyses stratified by gender or *APOE* ε4 status (performed only when *P* for interaction <0.10 in the main analyses, as shown in Table 3).

Food Intake (Score 0–4)	Incidence of AD (Model 2)		Memory Decline (Model 2)	
	*HR* (95% CI)	*P*	*B* (95% CI)	*P*
By gender				
Olive oil				
Men (*n* = 910)			0.06 (−0.05; 0.16)	0.285
Women (*n* = 1712)			−0.08 (−0.16; 0.01)	0.065
Red wine				
Men (*n* = 910)	0.82 (0.74; 0.92)	<0.001		
Women (*n* = 1712)	1.15 (1.00; 1.32)	0.044		
White wine				
Men (*n* = 910)			0.04 (−0.09; 0.17)	0.562
Women (*n* = 1712)			−0.13 (−0.26; 0.001)	0.052
By *APOE* ε4 status				
Fruits and vegetables				
*APOE* ε4 carrier (*n* = 552)	1.29 (0.73; 2.27)	0.388		
*APOE* ε4 non-carrier (*n* = 2070)	1.17 (0.88; 1.55)	0.287		
Meat and sausages				
*APOE* ε4 carrier (*n* = 552)	1.13 (0.90; 1.42)	0.293		
*APOE* ε4 non-carrier (*n* = 2070)	1.04 (0.87; 1.22)	0.615		
White wine				
*APOE* ε4 carrier (*n* = 552)	1.21 (1.01; 1.46)	0.044		
*APOE* ε4 non-carrier (*n* = 2070)	0.93 (0.82; 1.06)	0.245		
Coffee				
*APOE* ε4 carrier (*n* = 552)			0.11 (−0.06; 0.29)	0.202
*APOE* ε4 non-carrier (*n* = 2070)			−0.04 (−0.11; 0.02)	0.211

Based on imputed data (*n* = 2622). Model 2 was adjusted for age, BMI, education, smoking status, physical activity score, depression, hypercholesterolemia, modified physical comorbidity (CCI score) and *APOE* ε4 status (for the gender-stratified analyses) or gender (for the *APOE* ε4 stratified analyses). *P* < 0.05 was considered statistically significant. Abbreviations: AD, Alzheimer´s dementia; *APOE* ε4, apolipoprotein E ε4 allele; HR, hazard ratio; JM, joint modelling.

## Supplementary Materials

The following are available online at http://www.mdpi.com/2072-6643/10/7/852/s1, Table S1: Participant characteristics (original data) of the AgeCoDe cohort at follow-up 1 (*n* = 2622), Table S2: Specification of the multiple imputation procedure, Table S3: Longitudinal JM associations between food intake and or incident AD and memory decline over a 10-year follow-up period.

## References

[1] Blennow K., de Leon M.J., Zetterberg H. (2006). Alzheimer’s disease. Lancet.

[2] Jicha G.A., Markesbery W.R. (2010). Omega-3 fatty acids: Potential role in the management of early Alzheimer’s disease. Clin. Interv. Aging.

[3] Brookmeyer R., Johnson E., Ziegler-Graham K., Michael Arrighiet H. (2007). Forecasting the global burden of Alzheimer’s disease. Alzheimers Dement.

[4] Williams J.W., Plassman B.L., Burke J., Holsinger T., Benjamin S. (2010). Preventing Alzheimer’s disease and cognitive decline. Evid. Rep. Technol. Assess..

[5] Bell I.R. (2005). Diet and nutrition in Alzheimer’s disease and other dementias of late life. Explore.

[6] Hu N., Yu J.T., Tan L., Wang Y.L., Sun L., Tan L. (2013). Nutrition and the risk of Alzheimer’s disease. Biomed. Res. Int..

[7] Solfrizzi V., Panza F., Frisardi V., Seripa D., Logroscino G., Imbimbo B.P., Pilotto A. (2011). Diet and Alzheimer’s disease risk factors or prevention: The current evidence. Expert Rev. Neurother..

[8] Parletta N., Milte C.M., Meyer B.J. (2013). Nutritional modulation of cognitive function and mental health. J. Nutr. Biochem..

[9] Mi W., van Wijk N., Cansev M., Sijben J.W., Kamphuis P.J. (2013). Nutritional approaches in the risk reduction and management of Alzheimer’s disease. Nutrition.

[10] Swaminathan A., Jicha G.A. (2014). Nutrition and prevention of Alzheimer’s dementia. Front. Aging Neurosci..

[11] Otaegui-Arrazola A., Amiano P., Elbusto A., Urdaneta E., Martínez-Lage P. (2014). Diet, cognition, and Alzheimer’s disease: Food for thought. Eur. J. Nutr..

[12] Jacobs D.R., Steffen L.M. (2003). Nutrients, foods, and dietary patterns as exposures in research: A framework for food synergy. Am. J. Clin. Nutr..

[13] Jacobs D.R., Tapsell L.C. (2007). Food, not nutrients, is the fundamental unit in nutrition. Nutr. Rev..

[14] Cederholm T. (2017). Fish consumption and omega-3 fatty acid supplementation for prevention or treatment of cognitive decline, dementia or Alzheimer’s disease in older adults—Any news?. Curr. Opin. Clin. Nutr. Metab. Care.

[15] Arntzen K.A., Schirmer H., Wilsgaard T., Mathiesen E.B. (2010). Moderate wine consumption is associated with better cognitive test results: A 7 year follow up of 5033 subjects in the Tromso Study. Acta Neurol. Scand..

[16] Berr C., Portet F., Carriere I., Akbaraly T.N., Feart C., Gourlet V., Combe N., Barberger-Gateau P., Ritchie K. (2009). Olive oil and cognition: Results from the three-city study. Dement Geriatr. Cogn. Disord..

[17] Loef M., Walach H. (2012). Fruit, vegetables and prevention of cognitive decline or dementia: A systematic review of cohort studies. J. Nutr. Health Aging.

[18] Liu Q.P., Wu Y.F., Cheng H.Y., Xia T., Ding H., Wang H., Wang Z.M., Xu Y. (2016). Habitual coffee consumption and risk of cognitive decline/dementia: A systematic review and meta-analysis of prospective cohort studies. Nutrition.

[19] Mandel S.A., Youdim M.B. (2012). In the rush for green gold: Can green tea delay age-progressive brain neurodegeneration?. Recent Pat. CNS Drug Discov..

[20] Lim H.J., Shim S.B., Jee S.W., Lee S.H., Lim C.J., Hong J.T., Sheen Y.Y., Hwang D.Y. (2013). Green tea catechin leads to global improvement among Alzheimer’s disease-related phenotypes in NSE/hAPP-C105 Tg mice. J. Nutr. Biochem..

[21] Noguchi-Shinohara M., Yuki S., Dohmoto C., Ikeda Y., Samuraki M., Iwasa K., Yokogawa M., Asai K., Komai K., Nakamura H. (2014). Consumption of green tea, but not black tea or coffee, is associated with reduced risk of cognitive decline. PLoS ONE.

[22] Albanese E., Dangour A.D., Uauy R., Acosta D., Guerra M., Guerra S.S.G., Huang Y.Q., Jacob K.S., Rodriguez J.L.d., Noriega L.H. (2009). Dietary fish and meat intake and dementia in Latin America, China, and India: A 10/66 Dementia Research Group population-based study. Am. J. Clin. Nutr..

[23] Giem P., Beeson W.L., Fraser G.E. (1993). The incidence of dementia and intake of animal products: Preliminary findings from the Adventist Health Study. Neuroepidemiology.

[24] Barberger-Gateau P., Letenneur L., Deschamps V., Pérès K., Dartigues J.F., Renaud S. (2002). Fish, meat, and risk of dementia: Cohort study. BMJ.

[25] Morris M.C., Evans D.A., Hebert L.E., Bienias J.L. (1999). Methodological issues in the study of cognitive decline. Am. J. Epidemiol..

[26] Nock T.G., Chouinard-Watkins R., Plourde M. (2017). Carriers of an apolipoprotein E epsilon 4 allele are more vulnerable to a dietary deficiency in omega-3 fatty acids and cognitive decline. Biochim. Biophys. Acta.

[27] Barberger-Gateau P., Samieri C., Féart C., Plourdeet M. (2011). Dietary omega 3 polyunsaturated fatty acids and Alzheimer’s disease: Interaction with apolipoprotein E genotype. Curr. Alzheimer Res..

[28] Smith P.J., Blumenthal J.A. (2016). Dietary Factors and Cognitive Decline. J. Prev. Alzheimers Dis..

[29] Morris M.C. (2016). Nutrition and risk of dementia: Overview and methodological issues. Ann. N. Y. Acad. Sci..

[30] Bunce D., Kivipelto M., Wahlin A. (2004). Utilization of cognitive support in episodic free recall as a function of apolipoprotein E and vitamin B12 or folate among adults aged 75 years and older. Neuropsychology.

[31] Huang T.L., Zandi P.P., Tucker K.L., Fitzpatrick A.L., Kuller L.H., Fried L.P., Burke G.L., Carlson M.C. (2005). Benefits of fatty fish on dementia risk are stronger for those without APOE epsilon4. Neurology.

[32] Martínez-Lapiscina E.H., Galbete C., Corella D., Toledo E., Buil-Cosiales P., Salas-Salvadó J., Ros E., Martínez-González M.Á. (2014). Genotype patterns at CLU, CR1, PICALM and APOE, cognition and Mediterranean diet: The PREDIMED-NAVARRA trial. Genes Nutr..

[33] Van de Rest O., Wang Y., Barnes L.L., Tangney C., Bennett D.A., Morriset M.C. (2016). APOE epsilon4 and the associations of seafood and long-chain omega-3 fatty acids with cognitive decline. Neurology.

[34] Tudorache I.F., Trusca V.G., Gafencu A.V. (2017). Apolipoprotein E—A Multifunctional Protein with Implications in Various Pathologies as a Result of Its Structural Features. Comput. Struct. Biotechnol. J..

[35] Zhao N., Liu C.C., Qiao W., Bu G. (2018). Apolipoprotein E, Receptors, and Modulation of Alzheimer’s Disease. Biol. Psychiatry.

[36] Wisniewski T., Frangione B. (1992). Apolipoprotein E: A pathological chaperone protein in patients with cerebral and systemic amyloid. Neurosci. Lett..

[37] Arab L., Biggs M.L., O’Meara E.S., Longstreth W.T., Crane P.K., Fitzpatrick A.L. (2011). Gender differences in tea, coffee, and cognitive decline in the elderly: The Cardiovascular Health Study. J. Alzheimers Dis..

[38] Lassek W.D., Gaulin S.J. (2011). Sex differences in the relationship of dietary Fatty acids to cognitive measures in american children. Front. Evol. Neurosci..

[39] Araújo L.F., Mirza S.S., Bos D., Niessen W.J., Barreto S.M., van der Lugt A., Vernooij M.W., Hofman A., Tiemeier H., Ikram M.A. (2016). Association of Coffee Consumption with MRI Markers and Cognitive Function: A Population-Based Study. J. Alzheimers Dis..

[40] Tsiatis A.A., Davidian M. (2004). Joint Modeling of longitudinal and time-to-event data: An overview. Stat. Sin..

[41] Ibrahim J.G., Chu H., Chen L.M. (2010). Basic concepts and methods for joint models of longitudinal and survival data. J. Clin. Oncol..

[42] Sudell M., Kolamunnage-Dona R., Tudur-Smith C. (2016). Joint models for longitudinal and time-to-event data: A review of reporting quality with a view to meta-analysis. BMC Med. Res. Methodol..

[43] Asar O., Ritchie J., Kalra P.A., Diggle P.J. (2015). Joint modelling of repeated measurement and time-to-event data: An introductory tutorial. Int. J. Epidemiol..

[44] Luck T., Riedel-Heller S.G., Kaduszkiewicz H., Bickel H., Jessen F., Pentzek M., Wiese B., Koelsch H., van den Bussche H., Abholz H.H. (2007). Mild cognitive impairment in general practice: Age-specific prevalence and correlate results from the German study on ageing, cognition and dementia in primary care patients (AgeCoDe). Dement Geriatr. Cogn. Disord..

[45] Jessen F., Wiese B., Bickel H., Eiffländer-Gorfer S., Fuchs A., Kaduszkiewicz H., Köhler M., Luck T., Mösch E., Pentzek M. (2011). Prediction of dementia in primary care patients. PLoS ONE.

[46] Cooper B., Bickel H., Schaufele M. (1992). The ability of general-practitioners to detect dementia and cognitive impairment in their elderly patients—A study in Mannheim. Int. J. Geriatr. Psychiatry.

[47] Zaudig M., Hiller W. (1996). SIDAM-Handbuch Strukturiertes Interview für die Diagnose einer Demenz vom Alzheimer Typ, der Multiinfarkt- (Oder Vaskulären) Demenz und Demenzen Anderer Ätiologie nach DSM-III-R, DSM-IV, ICD-10.

[48] Zaudig M., Mittelhammer J., Hiller W., Pauls A., Thora C., Morinigo A., Mombour W. (1991). SIDAM—A structured interview for the diagnosis of dementia of the Alzheimer type, multi-infarct dementia and dementias of other aetiology according to ICD-10 and DSM-III-R. Psychol. Med..

[49] McKhann G., Drachman D., Folstein M., Katzman R., Price D., Stadlanet E.M. (1984). Clinical diagnosis of Alzheimer’s disease: Report of the NINCDS-ADRDA Work Group under the auspices of Department of Health and Human Services Task Force on Alzheimer’s Disease. Neurology.

[50] Román G.C., Tatemichi T.K., Erkinjuntti T., Cummings J.L., Masdeu J.C., Garcia J.H., Amaducci L., Orgogozo J.M., Brun A., Hofman A. (1993). Vascular dementia: Diagnostic criteria for research studies. Report of the NINDS-AIREN International Workshop. Neurology.

[51] Reisberg B., Ferris S.H., de Leon M.J., Crook T. (1982). The Global Deterioration Scale for assessment of primary degenerative dementia. Am. J. Psychiatry.

[52] Blessed G. (1996). The association between quantitative measures of dementia and of senile change in the cerebral grey matter of elderly subjects—Retrospective. Int. J. Geriatr. Psychiatry.

[53] Moms J.C., Heyman A., Mohs R.C., Hughes J.P., Belle G.v., Fillenbaum G., Mellits E.D., Clarket C. (1989). The Consortium to Establish a Registry for Alzheimer’s Disease (CERAD). Part I. Clinical and neuropsychological assessment of Alzheimer’s disease. Neurology.

[54] Brauns H., Steinmann S. (1999). Educational reform in France, West-Germany and the United Kingdom: Updating the CASMIN educational classification. ZUMA Nachr..

[55] Hixson J.E., Vernier D.T. (1990). Restriction isotyping of human apolipoprotein E by gene amplification and cleavage with HhaI. J. Lipid Res..

[56] Verghese J., Lipton R.B., Katz M.J., Hall C.B., Derby C.A., Kuslansky G., Ambrose A.F., Sliwinski M., Buschke H. (2003). Leisure activities and the risk of dementia in the elderly. N. Engl. J. Med..

[57] Sheikh J.I., Yesavage J.A., Brooks J.O., Friedman L., Gratzinger P., Hill R.D., Zadeik A., Crook T. (1991). Proposed factor structure of the Geriatric Depression Scale. Int. Psychogeriatr..

[58] Gauggel S., Birkner B. (1999). Birkner, Validity and reliability of a German version of the Geriatric Depression Scale (GDS). Z. Fur Klin. Psychol.-Forsch. Praxis.

[59] Charlson M.E., Pompei P., Ales K.L., MacKenzie C.R. (1987). A new method of classifying prognostic comorbidity in longitudinal studies: Development and validation. J. Chronic Dis..

[60] Caruana M., Cauchi R., Vassallo N. (2016). Putative Role of Red Wine Polyphenols against Brain Pathology in Alzheimer’s and Parkinson’s Disease. Front. Nutr..

[61] Vasanthi H.R., Parameswari R.P., DeLeiris J., Das D.K. (2012). Health benefits of wine and alcohol from neuroprotection to heart health. Front. Biosci..

[62] Basli A., Soulet S., Chaher N., Mérillon J.M., Chibane M., Monti J.P., Richard T. (2012). Wine polyphenols: Potential agents in neuroprotection. Oxid. Med. Cell. Longev..

[63] Granzotto A., Zatta P. (2014). Resveratrol and Alzheimer’s disease: Message in a bottle on red wine and cognition. Front. Aging Neurosci..

[64] Weyerer S., Schäufele M., Wiese B., Maier W., Tebarth F., van den Bussche H., Pentzek M., Bickel H., Luppa M., Riedel-Heller S.G. (2011). Current alcohol consumption and its relationship to incident dementia: Results from a 3-year follow-up study among primary care attenders aged 75 years and older. Age Ageing.

[65] Peters R., Peters J., Warner J., Beckett N., Bulpitt C. (2008). Alcohol, dementia and cognitive decline in the elderly: A systematic review. Age Ageing.

[66] Zuccalà G., Onder G., Pedone C., Cesari M., Landi F., Bernabei R., Cocchi A., Gruppo Italiano di Farmacoepidemiologia nell’Anziano Investigatorset (2001). Dose-related impact of alcohol consumption on cognitive function in advanced age: Results of a multicenter survey. Alcohol. Clin. Exp. Res..

[67] Beydoun M.A., Beydoun H.A., Gamaldo A.A., Teel A., Zonderman A.B., Wang F. (2014). Epidemiologic studies of modifiable factors associated with cognition and dementia: Systematic review and meta-analysis. BMC Public Health.

[68] Panza F., Frisardi V., Seripa D., Logroscino G., Santamato A., Imbimbo B.P., Scafato E., Pilotto A., Solfrizzi V. (2012). Alcohol consumption in mild cognitive impairment and dementia: harmful or neuroprotective?. Int. J. Geriatr. Psychiatry.

[69] Shukitt-Hale B., Miller M.G., Chu Y.F., Lyle B.J., Joseph J.A. (2013). Coffee, but not caffeine, has positive effects on cognition and psychomotor behavior in aging. Age.

[70] Higdon J.V., Frei B. (2006). Coffee and health: A review of recent human research. Crit. Rev. Food Sci. Nutr..

[71] Panza F., Solfrizzi V., Barulli M.R., Bonfiglio C., Guerra V., Osella A., Seripa D., Sabbà C., Pilotto A., Logroscino G. (2015). Coffee, tea, and caffeine consumption and prevention of late-life cognitive decline and dementia: A systematic review. J. Nutr. Health Aging.

[72] Dall’Igna O.P., Fett P., Gomes M.W., Souza D.O., Cunha R.A., Lara D.R. (2007). Caffeine and adenosine A(2a) receptor antagonists prevent beta-amyloid (25-35)-induced cognitive deficits in mice. Exp. Neurol..

[73] Eskelinen M.H., Ngandu T., Tuomilehto J., Soininen H., Kivipelto M. (2009). Midlife coffee and tea drinking and the risk of late-life dementia: A population-based CAIDE study. J. Alzheimers Dis..

[74] Lindsay J., Laurin D., Verreault R., Hébert R., Helliwell B., Hill G.B., McDowell I. (2002). Risk factors for Alzheimer’s disease: A prospective analysis from the Canadian Study of Health and Aging. Am. J. Epidemiol..

[75] Martínez-Lapiscina E.H., Clavero P., Toledo E., San Julián B., Sanchez-Tainta A., Corella D., Lamuela-Raventós R.M., Martínez J.A., Martínez-Gonzalez M.Á. (2013). Virgin olive oil supplementation and long-term cognition: The PREDIMED-NAVARRA randomized, trial. J. Nutr. Health Aging.

[76] Zhang Y., Chen J., Qiu J., Li Y., Wang J., Jiao J. (2016). Intakes of fish and polyunsaturated fatty acids and mild-to-severe cognitive impairment risks: A dose-response meta-analysis of 21 cohort studies. Am. J. Clin. Nutr..

[77] Miller M.G., Thangthaeng N., Poulose S.M., Shukitt-Hale B. (2017). Role of fruits, nuts, and vegetables in maintaining cognitive health. Exp. Gerontol..

[78] Yusufov M., Weyandt L.L., Piryatinsky I. (2017). Alzheimer’s disease and diet: A systematic review. Int. J. Neurosci..

[79] Granic A., Davies K., Adamson A., Kirkwood T., Hill T.R., Siervo M., Mathers J.C., Jagger C. (2016). Dietary Patterns High in Red Meat, Potato, Gravy, and Butter Are Associated with Poor Cognitive Functioning but Not with Rate of Cognitive Decline in Very Old Adults. J. Nutr..

[80] Fang X., An P., Wang H., Wang X., Shen X., Li X., Min J., Liu S., Wang F. (2015). Dietary intake of heme iron and risk of cardiovascular disease: A dose-response meta-analysis of prospective cohort studies. Nutr. Metab. Cardiovasc. Dis..

[81] Jakobsen L.H., Kondrup J., Zellner M., Tetens I., Roth E. (2011). Effect of a high protein meat diet on muscle and cognitive functions: A randomised controlled dietary intervention trial in healthy men. Clin. Nutr..

[82] Azhar Z.M., Zubaidah J.O., Norjan K.O.N., Zhuang C.Y.J., Fai T. (2013). A pilot placebo-controlled, double-blind, and randomized study on the cognition-enhancing benefits of a proprietary chicken meat ingredient in healthy subjects. Nutr. J..

[83] Fotuhi M., Mohassel P., Yaffe K. (2009). Fish consumption, long-chain omega-3 fatty acids and risk of cognitive decline or Alzheimer disease: A complex association. Nat. Clin. Pract. Neurol..

[84] Daiello L.A., Gongvatana A., Dunsiger S., Cohen R.A., Ottet B.R. (2015). Association of fish oil supplement use with preservation of brain volume and cognitive function. Alzheimers Dement.

[85] Salem N., Vandal M., Calon F. (2015). The benefit of docosahexaenoic acid for the adult brain in aging and dementia. Prostaglandins Leukot Essent Fat. Acids.

[86] Ng T.P., Feng L., Niti M., Kua E.H., Yap K.B. (2008). Tea consumption and cognitive impairment and decline in older Chinese adults. Am. J. Clin. Nutr..

[87] Venkatesan R., Ji E., Kim S.Y. (2015). Phytochemicals that regulate neurodegenerative disease by targeting neurotrophins: A comprehensive review. Biomed. Res. Int..

[88] Mandel S.A., Weinreb O., Amit T., Youdim M.B. (2012). Molecular mechanisms of the neuroprotective/neurorescue action of multi-target green tea polyphenols. Front. Biosci..

[89] Dietz C., Dekker M. (2017). Effect of Green Tea Phytochemicals on Mood and Cognition. Curr. Pharm. Des..

[90] Rains T.M., Agarwal S., Maki K.C. (2011). Antiobesity effects of green tea catechins: A mechanistic review. J. Nutr. Biochem..

[91] Rothman K.J. (2002). Epidemiology—An Introduction.

[92] Schröder H., Fitó M., Estruch R., Martinez-González M.Z., Corella D., Salas-Salvadó J., Lamuela-Raventós R., Ros E., Salaverría I., Fiol M. (2011). A short screener is valid for assessing Mediterranean diet adherence among older Spanish men and women. J. Nutr..

[93] Volkert D., Schrader E. (2013). Dietary assessment methods for older persons: What is the best approach?. Curr. Opin. Clin. Nutr. Metab. Care.

[94] Block G., Gillespie C., Rosenbaum E.H., Jenson C. (2000). A rapid food screener to assess fat and fruit and vegetable intake. Am. J. Prev. Med..

[95] Topiwala A., Allan C.L., Valkanova V., Zsoldos E., Filippini N., Sexton C., Mahmood A., Fooks P., Singh-Manoux A., Mackay C.E. (2017). Moderate alcohol consumption as risk factor for adverse brain outcomes and cognitive decline: Longitudinal cohort study. BMJ.

[96] Morris M.C., Evans D.A., Tangney C.C., Bienias J.L., Wilson R.S. (2006). Associations of vegetable and fruit consumption with age-related cognitive change. Neurology.

