# Supplementary material for: Prospective Associations between Single Foods, Alzheimer’s Dementia and Memory Decline in the Elderly
Source: Nutrients. 2018 Jun 29;10(7):852. doi: 10.3390/nu10070852 (PMC6073331; doi:10.3390/nu10070852)
Supplement: Supplementary file 1 [file nutrients-10-00852-s001.pdf]

SUPPLEMENTARY TABLES

Table S1. Participant characteristics (original data) of the AgeCoDe cohort at follow-up 1 (*n* = 2622).

**Participants characteristics**	**Total population (*n* = 2622)**	**Men (*n* = 910)**	**Women (*n* =1712)**	***P***	***APOE* ε4 non-carriers (*n* =2004)**	***APOE* ε4 carriers (n = 524)**	***P***
Age (years)	81.2 ± 3.4	80.9 ± 3.4	81.3 ± 3.5	0.001	81.2 ± 3.5	80.8 ± 3.3	0.021
Female (*n* (%))	1,712 (65.3)	-	-	-	1,305 (65.1)	337 (64.3)	0.730
Height in cm	165± 7	170± 7	162± 6	<0.001	164 ± 7	165 ± 7	0.208
**Weight in kg**	70± 11	76± 11	67±10	<0.001	70 ±11	70 ±10	0.346
Missing values	*4*	*0*	*4*		*4*	*0*	
***APOE* ε4 allele** (*n* (%))	524 (20.0)	187 (20.5)	337 (19.7)	0.730	-	-	-
Missing values	*94*	*24*	*70*				
Education (*n* (%))				<0.001			0.255
Low	1,594 (60.8)	483 (53.1)	1,111 (64.9)		1,226 (61.2)	312 (59.5)	
Middle	723 (27.6)	220 (24.2)	503 (29.4)		535 (26.7)	157 (30.0)	
High	305 (11.6)	207 (22.7)	98 (5.7)		243 (12.1)	55 (10.5)	
**Physical activity** (*n* (%))				0.530			0.186
Low (0-<3)	830 (31.7)	276 (30.3)	554 (32.4)		652 (32.5)	153 (29.2)	
Middle (3-<5)	893 (34.1)	295 (32.4)	598 (34.9)		668 (33.3)	195 (37.2)	
High (5-11)	889 (33.9)	337 (37.0)	552 (32.2)		677 (33.8)	173 (33.0)	
Missing values	*10*	*2*	*8*		*7*	*3*	
Smoking (*n* (%))				0.106			0.488
Never	1,307 (49.8)	178 (19.6)	1,129 (66.0)		991 (49.5)	260 (49.6)	
Past	1,125 (42.9)	662 (72.7)	463 (27.0)		870 (43.4)	219 (41.8)	
Current	190 (7.3)	70 (7.7)	120 (7.0)		143 (7.1)	45 (8.6)	
MCI [n (%)]	436 (16.6)	121 (13.3)	315 (18.4)	0.001	304 (15.2)	113 (21.6)	<0.001
**Hypercholesterolemia (*n* (%))**	1,408 (53.7)	446 (49.0)	962 (56.2)	0.106	1,066 (53.2)	306 (58.4)	0.012
Missing values	*17*	*5*	*12*		*4*	*1*	
Depression (*n* (%))	298 (11.4)	80 (8.8)	218 (12.7)	0.002	222 (11.1)	64 (12.2)	0.465
**Modified CCI score**				<0.001			0.103
Score 0-2	1,811 (69.1)	569 (62.5)	1,242 (72.5)		1,368 (68.3)	379 (72.3)	
Score 3-4	635 (24.2)	274 (30.1)	361 (21.1)		504 (25.1)	108 (20.6)	
Score 5-6	82 (3.1)	38 (4.2)	44 (2.6)		63 (3.1)	16 (3.1)	
Missing values	*94*	*29*	*65*		*69*	*21*	
CERAD memory (score 0-100)	71.7 ± 13.0	69.3 ± 12.8	73.0 ± 13.0	<0,.001	72.4 ± 12.7	69.4 ± 13.7	<0.001
Time to develop AD dementia (years)	4.5 ± 2.8	4.2 ± 2.7	4.6 ± 2.8	0.133	4.2 ± 2.7	4.6 ± 2.8	0.183

Time to censoring (years)	5.9 ± 3.3	5.7 ± 3.3	6.0 ± 3.3	0.047	5.6 ± 3.3	6.0 ± 3.3	0.021

Data (n = 2622) are means (± standard deviation) or n (%). $P < 0.05$ was considered statistically significant. Abbreviations; AgeCoDe, German Study on Aging, Cognition and Dementia in Primary Care Patients; *APOE* ε4 , apolipoprotein E ε4 allele; MCI, mild cognitive impairment; CCI, Charlson comorbidity index; CERAD; Consortium to Establish a Registry for Alzheimer's Disease; AD, Alzheimer´s dementia.

Table S2. Specification of the multiple imputation procedure.

**Software**	**IBM SPSS Statistics for Windows (Release 21)**
Imputation method	Fully conditional specification (Markov chain Monte Carlo method)
Key settings	Maximum iterations: 20
Imputed data sets	10
Variables included in the imputation procedure (imputed or used as predictors of missing data):	olive oil, fresh fish, red wine, white wine, green tea, coffee, meat and sausages, fruits and vegetables, memory score FU1-FU8, Visit FU1-FU8, incidence of dementia FU1-FU8, incidence of Alzheimer's dementia (AD) FU1-FU8, incidence of vascular dementia FU1-FU8, duration to develop (AD) dementia FU1-FU8, cognitive health status (cognitively normal, MCI, overall dementia), age, gender, *APOE* ε4, education (baseline), height (cm), weight (kg), physical activity score, smoking, hypercholesterolemia, depression, myocardial infarction, congestive heart failure, peripheral vascular diseases, cerebro vascular diseases, rheumatism (baseline), diabetes, chronic kidney diseases, liver diseases.
Additionally added predictive variables to increase plausibility of missing at random assumption:	medication FU1, housekeeping FU1, diabetes (baseline), cardiovascular diseases (baseline), renal insufficiency (baseline), hypercholesterolemia (baseline), liver diseases (baseline)
Not normally distributed variables were treated with:	Predictive mean matching
Binary/categorical variables were treated with:	Logistic regression models

Abbreviations: FU, follow up; MCI, mild cognitive impairment; *APOE* ε4, apolipoprotein E ε4 allele.

Table S3. Longitudinal JM associations between food intake and or incident AD and memory decline over a 10-year follow-up period

**Associations between Food Intake and Incident AD or Memory Decline**	**HR (95% CI) for incident AD and unstandardized regression coefficients (95% CI) for memory decline**			
	**Model 1**		**Model 2**	
Incident AD				
(survival sub-model)	*HR* (95%CI)	*P*	*HR* (95%CI)	*P*
Fruits and vegetables	1.04 (0.78; 1.39)	0.786	1.08 (0.80; 1.46)	0.609
Fresh fish	0.95 (0.84; 1.07)	0.375	0.98 (0.87; 1.11)	0.754
Olive oil	0.98 (0.91; 1.05)	0.497	1.00 (0.93; 1.07)	0.969
Meat and sausages	1.08 (0.94; 1.25)	0.270	1.09 (0.94; 1.26)	0.236
Red wine	0.91 (0.84; 0.98)	0.018	0.92 (0.85; 0.99)	0.045
White wine	0.97 (0.88; 1.08)	0.645	1.00 (0.91; 1.12)	0.875
Coffee	0.96 (0.90; 1.03)	0.240	0.97 (0.90; 1.04)	0.338
Green tea	0.91 (0.84; 0.99)	0.039	0.94 (0.86; 1.02)	0.129
Memory decline				
(repeated-measures sub-model)	*B* (95%CI)	*P*	*B* (95%CI)	*P*
Fruits and vegetables	0.10 (-0.13; 0.34)	0.386	0.10 (-0.14; 0.33)	0.408
Fresh fish	-0.03 (-0.14; 0.08)	0.602	-0.03 (-0.14; 0.08)	0.610
Olive oil	-0.03 (-0.09; 0.04)	0.385	-0.03 (-0.09; 0.04)	0.388
Meat and sausages	0.01 (-0.12; 0.13)	0.893	0.01 (-0.11; 0.14)	0.845
Red wine	-0.04 (-0.11; 0.03)	0.308	-0.04 (-0.11; 0.03)	0.302
White wine	-0.03 (-0.12; 0.06)	0.520	-0.03 (-0.12; 0.06)	0.494
Coffee	-0.02 (-0.08; 0.05)	0.148	-0.02 (-0.08; 0.05)	0.241
Green tea	0.02 (-0.06; 0.09)	0.676	0.02 (-0.06; 0.09)	0.681

Based on imputed data (n = 2622).
Model 1is adjusted for age, gender, BMI, education and *APOE* ε4 carrier status.
Model 2 is adjusted as for model 1, plus smoking status, physical activity score, depression, hypercholesterolemia, and a modified CCI score.
$P < 0.05$ was considered statistically significant.
Abbreviations: JM, joint modelling; HR, hazard ratio; AD, Alzheimer's dementia; BMI, body mass index; *APOE* ε4 , apolipoprotein E ε4 allele.
